# Enzyme-Based Anti-Inflammatory Therapeutics for Inflammatory Diseases

**DOI:** 10.3390/pharmaceutics17050606

**Published:** 2025-05-02

**Authors:** Kannan Badri Narayanan

**Affiliations:** 1 School of Chemical Engineering, Yeungnam University, 280 Daehak-Ro, Gyeongsan 38541, Gyeongbuk, Republic of Korea; okbadri@gmail.com or okbadri@yu.ac.kr; 2 Research Institute of Cell Culture, Yeungnam University, 280 Daehak-Ro, Gyeongsan 38541, Gyeongbuk, Republic of Korea

**Keywords:** inflammation, therapeutic enzymes, pro-inflammatory mediators, oxidoreductases, hydrolases, protease, engineered enzymes

## Abstract

Inflammation is a multifaceted biological response of the immune system against various harmful stimuli, including pathogens (such as bacteria and viruses), cellular damage, toxins, and natural/synthetic irritants. This protective mechanism is essential for eliminating the cause of injury, removing damaged cells, and initiating the repair process. While inflammation is a fundamental component of the body’s defense and healing process, its dysregulation can lead to pathological consequences, contributing to various acute and chronic diseases, such as autoimmune disorders, cancer, metabolic syndromes, cardiovascular diseases, neurodegenerative conditions, and other systemic complications. Generally, non-steroidal anti-inflammatory drugs (NSAIDs), corticosteroids, disease-modifying anti-rheumatic drugs (DMARDs), antihistamines, biologics, and colchicine are used as pharmacological agents in the management of inflammatory diseases. However, these conventional treatments often have limitations, including adverse side effects, long-term toxicity, and drug resistance. In contrast, enzyme-based therapeutics have emerged as a promising alternative due to their high specificity, catalytic efficiency, and ability to modulate inflammatory pathways with reduced side effects. These enzymes function by scavenging reactive oxygen species (ROS), inhibiting cytokine transcription, degrading circulating cytokines, and blocking cytokine release by targeting exocytosis-related receptors. Additionally, their role in tissue repair and regeneration further enhances their therapeutic potential. Most natural anti-inflammatory enzymes belong to the oxidoreductase class, including catalase and superoxide dismutase, as well as hydrolases such as trypsin, chymotrypsin, nattokinase, bromelain, papain, serratiopeptidase, collagenase, hyaluronidase, and lysozyme. Engineered enzymes, such as Tobacco Etch Virus (TEV) protease and botulinum neurotoxin type A (BoNT/A), have also demonstrated significant potential in targeted anti-inflammatory therapies. Recent advancements in enzyme engineering, nanotechnology-based enzyme delivery, and biopharmaceutical formulations have further expanded their applicability in treating inflammatory diseases. This review provides a comprehensive overview of both natural and engineered enzymes, along with their formulations, used as anti-inflammatory therapeutics. It highlights improvements in stability, efficacy, and specificity, as well as minimized immunogenicity, while discussing their mechanisms of action and clinical applications and potential future developments in enzyme-based biomedical therapeutics.

## 1. Introduction

Enzymes are biocatalysts that regulate various metabolic biochemical reactions under physiological conditions in the human body [1]. They play a pivotal role in both the diagnosis and treatment of a wide range of diseases and disorders. According to the International Union of Biochemistry and Molecular Biology (IUBMB), enzymes are classified into seven functional classes: hydrolases, oxidoreductases, isomerases, lyases, transferases, ligases, and translocases [2,3]. Since the 19th century, therapeutic enzymes have been used as digestive aids, and their applications have expanded significantly to include treatments for numerous medical conditions. Several kinds of enzymes belonging to the class of proteases, lipases, nucleases, glucosidases, and kinases have gained considerable importance in the pharmaceutical industry due to their ability to catalyze specific biochemical reactions with high efficiency and selectivity [4,5]. Measuring enzyme levels in extracellular body fluids is also an important tool for clinical diagnosis and treatment of the disease. A small change in enzyme concentrations can be quantified accurately through enzyme assays for the detection of diseases. Unlike other clinical biochemical proteins such as albumin or globulin, enzyme biomarkers offer superior organ and disease specificity. Recent advancements in biosensor technology have further enhanced enzyme-based diagnostics, with enzyme electrodes integrated into potentiometric and amperometric detection systems, enabling highly sensitive and rapid analyte detection. For instance, cholesterol oxidase is employed to measure serum cholesterol levels, while glucose oxidase and catalase are used to quantify glucose in blood [6].

The use of enzymes in medicine and pharmacology has emerged as one of the most promising therapeutic strategies, offering targeted, efficient, and site-specific treatment approaches with commercial viability [7]. Their high substrate specificity enables precise modulation of metabolic and physiological processes, restoring cellular homeostasis. For optimal enzymatic function at minimal substrate concentrations, favorable kinetic properties, such as a low Michaelis constant (Km) and high maximum velocity (Vmax), are essential [8]. However, despite their advantages, therapeutic enzyme applications are often constrained by challenges such as high production costs, limited stability, immune responses, and a short in vivo half-life. To overcome these limitations, recombinant DNA technology has been employed to produce high-purity enzymes free of contaminants. These recombinant enzymes are expressed in genetically engineered bacterial, yeast, plant, mammalian, and insect cell systems, enhancing their therapeutic efficacy and commercial scalability [9,10].

Currently, therapeutic enzymes are widely utilized in diverse medical applications, including antimicrobial, anti-inflammatory, anti-cancer, and anticoagulant therapies, as well as in the treatment of fibrinolysis, metabolic storage disorders, and mucolytic conditions [11]. The global market for therapeutic enzymes was valued at USD 7322.4 million in 2023 and is projected to reach USD 16,750 million by 2030, with a Compound Annual Growth Rate (CAGR) of 12.6% [12]. These enzymes are administered through various delivery routes, including oral, injectable, and topical formulations, either independently or in combination with other therapies, for the treatment of various diseases.

Therapeutic enzymes represent a crucial frontier in modern medicine, offering a diverse array of treatment options for various diseases. Each enzyme, characterized by its unique catalytic properties, plays a distinct role in treating specific ailments, ranging from life-threatening disorders, such as acute lymphoblastic leukemia (ALL), to more prevalent ailments such as digestive disorders. Therapeutic enzymes utilized in disease treatments are derived from a diverse array of biological sources, including microorganisms, plants, and animals. Microbial-derived enzymes are particularly significant in clinical applications. For example, L-asparaginase, an enzyme used in ALL treatment, is primarily extracted from bacteria such as *Bacillus megaterium*, *Escherichia coli*, and *Erwinia chrysanthemi* [13,14,15], as well as fungi such as the agaricomycete Ganoderma australe GPC191 [16]. Streptokinase, derived from *Streptococcus* species such as *S. equinus* VIT_VB2 (isolated from bovine milk) and *S. dysgalactiae* subsp. equisimilis SK-6, has exhibited potent thrombolytic activity [17]. Collagenase, isolated from Clostridium histolyticum, is used for the removal of necrotic tissues and in the treatment of Dupuytren’s contracture [18,19]. Additionally, deoxyribonucleases (DNases), isolated from Escherichia coli, are utilized in the treatment of the genetic disorder cystic fibrosis, where they degrade extracellular DNA in mucus, as well as in autoimmune diseases such as lupus [20,21]. Another widely used microbial enzyme is serratiopeptidase, produced by the *Serratia* species, which functions as an anti-inflammatory agent and a potent alternative to nonsteroidal anti-inflammatory drugs (NSAIDs) [22].

Fungal-derived therapeutic enzymes have also gained much importance in recent times. For example, glucocerebrosidase, which is expressed in genetically engineered humanized Pichia pastoris, is used in the treatment of Gaucher’s disease, a metabolic disorder caused by glucocerebroside accumulation [23,24]. Lipases from Candida rugosa and Rhizomucor miehei have been used in the treatment of metabolic disorders and lifestyle diseases [25]. Plant-derived therapeutic enzymes include papain from Carica papaya, bromelain from Ananas comosus, and ficin from Ficus carica, which are widely used as anti-inflammatory agents, for treating digestive disorders, and for the removal of necrotic tissue [26,27]. Additionally, animal-derived enzymes such as pancreatin, elastase, trypsin, and chymotrypsin are primarily extracted from the porcine or bovine pancreas and are extensively used in the treatment of exocrine pancreatic insufficiency (EPI), as digestive aids, and in wound-healing therapies [28,29]. Recent advances in recombinant DNA technology, directed evolution, and enzyme immobilization techniques have revolutionized the field of therapeutic enzymes, enabling large-scale, cost-effective production of highly specific and efficient enzyme-based therapeutics. This review provides a comprehensive discussion of the therapeutic enzymes used in inflammatory disease treatment, elucidating their mechanism of action, signaling pathways, and recent advancements in biopharmaceutical formulations.

## 2. Therapeutic Enzymes

Enzymes are widely used in biocatalysis and therapeutics due to their high specificity and efficiency in catalyzing biochemical reactions in the human body. These enzymes play a critical role in various physiological and pathological processes and have been harnessed for therapeutic applications. The advent of recombinant DNA technology has enabled the large-scale production of enzymes with high purity and efficiency, making them indispensable in the biopharmaceutical industry. Therapeutic enzymes are employed in the synthesis of biomolecules, including hormones, antibodies, and vaccines, which are fundamental for disease prevention and treatment [30]. Due to their substrate specificity, enzymes selectively act on target molecules without disrupting other biological pathways, thereby enhancing therapeutic efficacy while minimizing off-target effects [31]. Enzyme-based therapies have been particularly successful in the treatment of genetic disorders caused by enzyme deficiencies or malfunctioning. For example, enzyme replacement therapy (ERT) has revolutionized the management of lysosomal storage diseases such as Gaucher’s disease, in which hydrolytic lysosomal β-glucocerebroside is administered to restore normal metabolic function, and Pompe disease, in which acid alpha-glucosidase (GAA) supplementation prevents glycogen accumulation in muscle tissues [32]. Another widely used enzyme-based therapy is tissue plasminogen activator (tPA), which is administered for the dissolution of blood clots in ischemic stroke and myocardial infarction patients, significantly improving patient outcomes by restoring blood flow [33]. In addition to direct therapeutic applications, enzymes also play a crucial role in drug metabolism, prodrug activation, and detoxification. For instance, the cytochrome P450 enzyme facilitates the biotransformation of drugs and toxins, ensuring their effective clearance from the body [34].

Conversely, enzyme inhibitors have been developed to modulate enzyme activity for therapeutic purposes. Protease inhibitors such as ritonavir and saquinavir target viral protease in HIV treatment, preventing viral maturation and replication, and thus slowing disease progression [35]. The emergence of enzyme-based gene-editing technologies, such as CRISPR-Cas9, has further expanded the therapeutic potential of enzymes. By enabling precise genetic modifications, CRISPR-based approaches hold promise for treating inherited disorders and developing personalized medicine strategies [36]. Moreover, enzymes serve as critical biomarkers for disease diagnosis and monitoring. Elevated levels of alanine aminotransferase (ALT) and aspartate aminotransferase (AST) in blood serum are indicative of liver inflammation and damage, providing valuable insights into hepatic function and disease progression [37]. Beyond their traditional applications, enzymes have shown immense potential in regulating inflammatory responses.

Certain enzymes modulate key signaling pathways involved in inflammation by degrading pro-inflammatory mediators or restoring homeostatic balance. For example, superoxide dismutase (SOD) and catalase (CAT) mitigate oxidative stress by neutralizing reactive oxygen species (ROS), thereby reducing inflammation-associated tissue damage [38]. Similarly, anti-inflammatory proteolytic enzymes, such as bromelain, papain, and serratiopeptidase, exhibit immunomodulatory effects by degrading inflammatory cytokines and fibrin deposits in affected tissues. These enzymes are increasingly being explored as standalone or adjunctive treatments for inflammatory diseases, offering a promising alternative to conventional NSAIDs and corticosteroids, which are often associated with adverse effects. The integration of enzyme-based therapeutics in modern medicine has significantly advanced the treatment of various diseases. These biologically active molecules exhibit high specificity and catalytic efficiency, making them ideal candidates for targeted therapy. However, the clinical translation of therapeutic enzymes faces several challenges, particularly regarding enzyme stability, bioavailability, and immunogenicity. To overcome these limitations, current research focuses on innovative formulation and delivery strategies. Approaches such as enzyme encapsulation, nanoparticle-based delivery systems, and PEGylation (polyethylene glycol modification) have shown promise in enhancing enzyme half-life, protecting enzymes from proteolytic degradation, and reducing immunogenicity [39].

Advancements in mammalian cell-based production systems have also enabled the generation of recombinant enzymes with more human-like post-translational modifications, improving their compatibility and function in therapeutics [40]. In addition, protein-engineering techniques such as rational design, directed evolution, and site-specific modifications have been employed to optimize enzyme activity, stability, and substrate specificity under physiological conditions [41]. Moreover, epitope engineering is also used to minimize the immune recognition of therapeutic enzymes, thereby reducing the risk of adverse immunogenic responses [42,43]. The development of antibody-enzyme conjugates (AECs) represents another novel strategy to enhance targeting specificity, improve bioavailability, and reduce off-target effects by directing enzymatic activity to the site of inflammation [44,45]. Therefore, collectively, all these interdisciplinary advancements are expected to significantly improve the therapeutic efficacy, safety, and clinical applicability of enzyme-based therapies for inflammatory diseases and other pathological conditions.

## 3. Biotechnological Strategies to Enhance Enzyme Therapeutics

Therapeutic enzymes represent a promising class of biologics that can serve as natural and more specific alternatives to synthetic drugs for treating various diseases. However, their broader clinical application is limited by several key challenges, including immunogenicity, limited shelf-life stability, suboptimal therapeutic efficiency, and difficulty in cost-effective scalability. Immunogenicity, particularly of enzymes derived from non-human sources, can elicit immune responses and the formation of neutralizing antibodies, thereby reducing therapeutic efficacy. To overcome these challenges, several strategies have been developed, including PEGylation, the use of human or humanized enzyme homologs, epitope engineering through site-directed mutagenesis, nanocarrier-based enzyme encapsulation, and co-administration with immunosuppressants. To address shelf-life stability, which is often compromised by environmental factors such as temperature, pH, and oxidant stress, stabilization techniques such as lyophilization, the incorporation of stabilizing excipients (sugars/polyols, amino acids, proteins, polymers, reducing agents, surfactants, cryoprotectants, antioxidants, chelators, buffers, and/or salts), bioconjugation, and protein engineering have been employed to enhance the enzyme’s thermal and structural stability. For example, PEGylation of superoxide dismutase (PEG-SOD; pegorgotein) significantly enhanced its thermal stability, circulatory half-life, and reduced antigenicity and immunogenicity [46].

To improve therapeutic efficiency, approaches such as targeted delivery systems, controlled-release formulations, and combination therapies with conventional anti-inflammatory drugs have been implemented to increase enzyme bioavailability and site-specific activity. For example, Padhy et al. [47] utilized protein engineering and bioconjugation to develop a novel lysosome-targeting β-glucosidase delivery system by covalently conjugating the enzyme with mannose-6-phosphate-functionalized glycopolypeptides (M6P-GP), enabling specific lysosomal trafficking. Similarly, Gui et al. [48] designed an antioxidative nanoparticle delivery system using SOD-loaded porous polymersome nanoparticles (SOD-NPs), which demonstrated prolonged retention and preferential accumulation in the synovium of murine knee joints. To enable cost-effective scalability, the use of high-yield recombinant expression systems, including prokaryotic hosts such as Escherichia coli, and eukaryotic platforms like yeast (*Pichia pastoris*), plants (*Nicotiana tabacum*, *Oryza sativa*, *Zea mays*), and mammalian cell lines (e.g., Chinese Hamster Ovary (CHO) cells), have been commonly utilized. These systems, when combined with optimized fermentation and downstream purification protocols, facilitate the large-scale production of enzyme therapeutics in a commercially viable manner. A notable example is Taliglucerase alfa, an enzyme replacement therapy for Gaucher disease produced using a carrot root cell culture-based plant expression system, representing a pioneering approach in plant cell-expressed biotherapeutics [49]. Together, these biotechnological interventions significantly enhance the therapeutic potential, stability, efficacy, and manufacturability of enzyme-based anti-inflammatory therapeutics.

## 4. Inflammation

Inflammation is a natural fundamental physiological response that enables the body to heal or recover from illness, injury, or exposure to physical, chemical, or biological agents [50]. While inflammation is essential for tissue repair and immune defense, dysregulated or persistent inflammation contributes to the pathogenesis of numerous chronic diseases. Inflammation is broadly categorized into two types: acute and chronic. Acute inflammation is the immune system’s immediate response to sudden injury or infection caused by external factors, typically lasting from a few hours to several days. This response is characterized by increased blood flow, prostaglandins, and cytokines. Examples of acute inflammation include bacterial infections like streptococcal pharyngitis (strep throat), viral infections that cause upper respiratory inflammation, and enteritis, which is inflammation of the small intestine due to bacterial or viral infections. On the other hand, chronic inflammation is a prolonged and sustained inflammatory response that can persist for months or even years, often leading to tissue damage and disease progression. It is implicated in over half of all global deaths and is associated with a wide range of conditions, including autoimmune diseases such as lupus and rheumatoid arthritis, where persistent inflammation causes significant joint and organ damage. Chronic inflammation is also linked to certain cancers, gastrointestinal disorders such as Crohn’s disease and inflammatory bowel disease (IBD), respiratory diseases like asthma and chronic obstructive pulmonary disease (COPD), metabolic disorders such as type 2 diabetes, and neurodegenerative conditions like Alzheimer’s and Parkinson’s diseases [51].

One of the most concerning consequences of chronic inflammation is its role in cancer development and progression. Chronic inflammatory processes contribute to tumorigenesis by promoting genetic mutations, epigenetic alterations, and a microenvironment conducive to cancer cell survival. Inflammatory cells release reactive oxygen species (ROS) and cytokines, which can induce DNA damage and drive oncogenic mutations [52]. While cancer is primarily caused by genetic mutations, environmental exposures (e.g., UV radiation, smoking), viral infections (e.g., human papilloma virus (HPV), hepatitis B and C viruses), and lifestyle factors (e.g., diet, obesity) also play critical roles in cancer initiation and progression [53]. The complex interplay between inflammation and cancer can result in both tumor-promoting and tumor-suppressing effects, influencing treatment outcomes. Enzymes such as matrix metalloproteinases (MMPs), DNA repair enzymes, and cyclooxygenases (COX-2) contribute to cancer progression by facilitating tumor invasion, angiogenesis, and immune evasion. Similarly, COX-1 and 2, lipoxygenases (LOX), nicotinamide adenine dinucleotide phosphate (NADPH) oxidases, nitric oxide synthases (NOS), and various proteases play significant roles in the inflammatory signaling cascades that drive disease pathophysiology [54].

**Figure 1:**
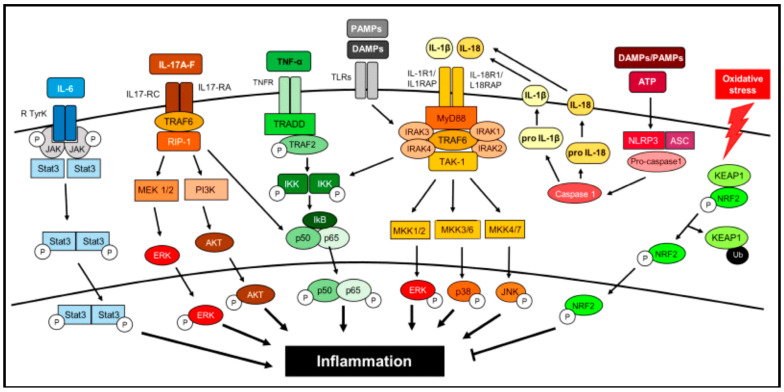
Intracellular signaling pathways in the activation of inflammatory pathways. Reproduced from [57], MDPI publishers.

The relationship between inflammation and therapeutic responses is intricate. While chronic inflammation often promotes tumor progression and resistance to treatment, acute inflammatory responses can enhance dendritic cell (DC) maturation and antigen presentation, thereby stimulating anti-tumor immune responses. This paradox highlights the need for therapeutic strategies that modulate inflammation in a disease-specific manner. A complex network of signaling pathways regulates inflammation initiation, progression, and resolution. The pathways include nuclear factor kappa B (NF-kB) signaling, which governs the expression of pro-inflammatory genes, the Janus kinase-signal transducer and activator of transcription (JAK-STAT) pathway, which modulates immune cell activation, Toll-like receptor (TLR) signaling, which detects microbial pathogens and endogenous danger signals, the cyclic GMP-AMP synthase/stimulator of the interferon genes (cGAS/STING) pathway, which is involved in antiviral immunity and inflammatory responses, and the mitogen-activated protein kinase (MAPK) cascade, which regulates cytokine production and cellular stress responses. Additionally, cytokines, chemokines, growth factors, inflammasomes, and inflammatory metabolites such as prostaglandins, leukotrienes, thromboxane, and specialized pro-resolving mediators (SPMs) play crucial roles in both inflammation and cancer progression, influencing therapeutic responses [55,56,57] (Figure 1).

**Figure 2:**
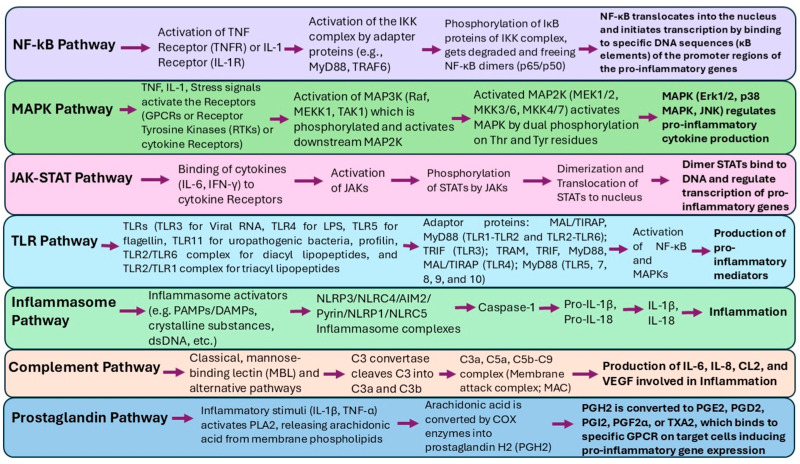
Key molecular signaling cascades involved in inflammatory pathways.

Another key aspect of inflammation regulation is the functional transformation of macrophages in inflammatory microenvironments. Macrophages exhibit plasticity and can switch between pro-inflammatory (M1) and anti-inflammatory (M2) phenotypes. M1 macrophages secrete pro-inflammatory cytokines such as tumor necrosis factor-alpha (TNF-α), interleukin-1 beta (IL-1β), and interleukin-6 (IL-6), which drive inflammation and immune responses against pathogens. In contrast, M2 macrophages are associated with tissue repair and the resolution of inflammation, releasing anti-inflammatory mediators such as interleukin-10 (IL-10), transforming growth factor-beta (TGF-β), and resolvins [58] (Figure 2). Understanding the molecular mechanisms underlying inflammation is essential for developing targeted enzyme-based anti-inflammatory therapeutics. Given the intricate role of enzymes in inflammatory processes, enzyme-based interventions have emerged as promising therapeutic strategies for inflammatory diseases.

## 5. Anti-Inflammatory Therapeutic Enzymes

Anti-inflammatory responses aim to mitigate inflammation, alleviate pain, and promote tissue healing. These responses can be evaluated using physiological and biochemical markers, which serve as indicators of inflammation severity and therapeutic effectiveness. The key biomarkers of inflammation include: elevated levels of C-reactive protein (CRP), a systemic inflammatory marker associated with cardiovascular disease, autoimmune disorders, and chronic inflammation; increased concentrations of pro-inflammatory cytokines, such as tumor necrosis factor-alpha (TNF-α), interleukin-6 (IL-6), and interleukin-1β (IL-1β) indicate ongoing inflammatory processes; higher erythrocyte sedimentation rate (ESR) and increased white blood cell count (WBC) suggest higher immune activation; and other inflammatory markers, such as serum amyloid A, fibrinogen, procalcitonin, and lipid peroxidation products (e.g., F1-isoprostanes, malondialdehyde), are associated with chronic inflammation and oxidative stress [59,60]. Beyond these biochemical markers, several key enzyme markers are also associated with inflammation: elevated levels of cyclooxygenases (COX-1 and COX-2) and lipoxygenase (LOX) contribute to the biosynthesis of inflammatory mediators such as prostaglandins and leukotrienes; there is increased activity of matrix metalloproteinases (MMPs), which degrade extracellular matrix (ECM) components, promoting tissue remodeling in inflammatory conditions; and there are reduced levels of antioxidant enzymes, such as superoxide dismutase (SOD) and glutathione peroxidase (GPx), which play protective roles by neutralizing ROS and mitigating oxidative damage [61,62]. The development of rheumatoid arthritis (RA) and osteoarthritis (OA) is closely linked to inflammatory mediators, including TNF-α, interleukins (ILs), synovial fibroblasts (fibroblast-like synoviocytes (FLS)), macrophages, and ROS produced by activated polymorphonuclear leukocytes [63].

Enzyme-based anti-inflammatory therapeutics target these inflammatory processes through three primary mechanisms: 1. scavenging ROS, in which enzymes neutralize ROS, thereby reducing oxidative stress and inhibiting the activation of inflammatory cytokines at the transcription level; 2. the direct degradation of circulating inflammatory cytokines by proteolytic enzymes, which breaks down pro-inflammatory cytokines, leading to reduced systemic inflammation; and 3. the prevention of cytokine release by the degradation of receptors involved in exocytosis, thereby inhibiting cytokine secretion and downstream inflammatory signaling. Most therapeutic anti-inflammatory natural enzymes belong to the enzyme classes oxidoreductases and hydrolases. Oxidoreductases facilitate oxidation-reduction reactions, helping to maintain redox balance and limit oxidative damage, whereas hydrolases catalyze the hydrolysis of various chemical bonds, aiding in the degradation of inflammatory mediators (Table 1).

**Figure 3:**
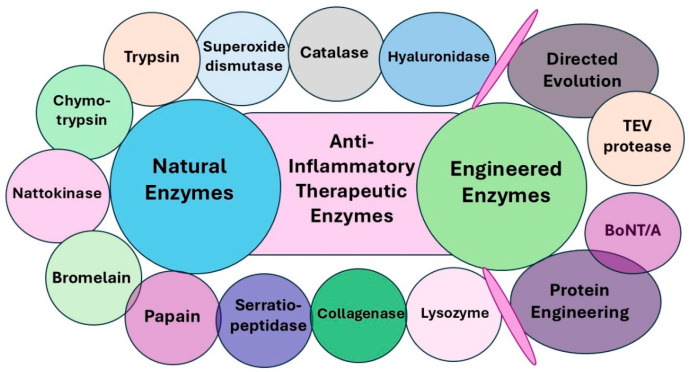
Various natural and engineered enzymes with anti-inflammatory therapeutic potential.

Moreover, the recent advancements in protein engineering and directed evolution have led to the development of engineered enzyme variants such as Tobacco Etch Virus (TEV) protease and botulinum neurotoxin type A (BoNT/A) with enhanced specificity and efficacy in inflammation modulation. These engineered enzymes also hold significant therapeutic potential in the treatment of chronic inflammatory diseases by interrupting inflammatory cascades, reducing tissue damage, and enhancing the resolution of inflammation. Further research into enzyme-based interventions may pay the way for targeted, biologically derived anti-inflammatory therapeutics with improved specificity and safety profiles (Figure 3).

## 6. Anti-Inflammatory Natural Enzymes

### 6.1. Oxidoreductases

Oxidoreductases are a class of enzymes that catalyze redox reactions in living organisms. This group includes oxidases, oxygenases, peroxidases, and dehydrogenases. Most oxidoreductase enzymes utilize cofactors such as nicotinamide adenine dinucleotide (NAD), flavin adenine dinucleotide (FAD), or nicotinamide adenine dinucleotide phosphate (NADP) to catalyze the redox reactions. The catalyzed reaction of oxidoreductases is as follows:

A^−^ + B → A + B^−^ (1)

where A is the oxidant (electron acceptor) and B is the reductant (electron donor), and in which the enzyme catalyzes the transfer of electrons from oxidant to reductant [100]. These enzymes play a crucial role in regulating oxidative stress, inflammation, and immune responses (Table 1).

#### 6.1.1. Catalase (CAT)

**Figure 4:**
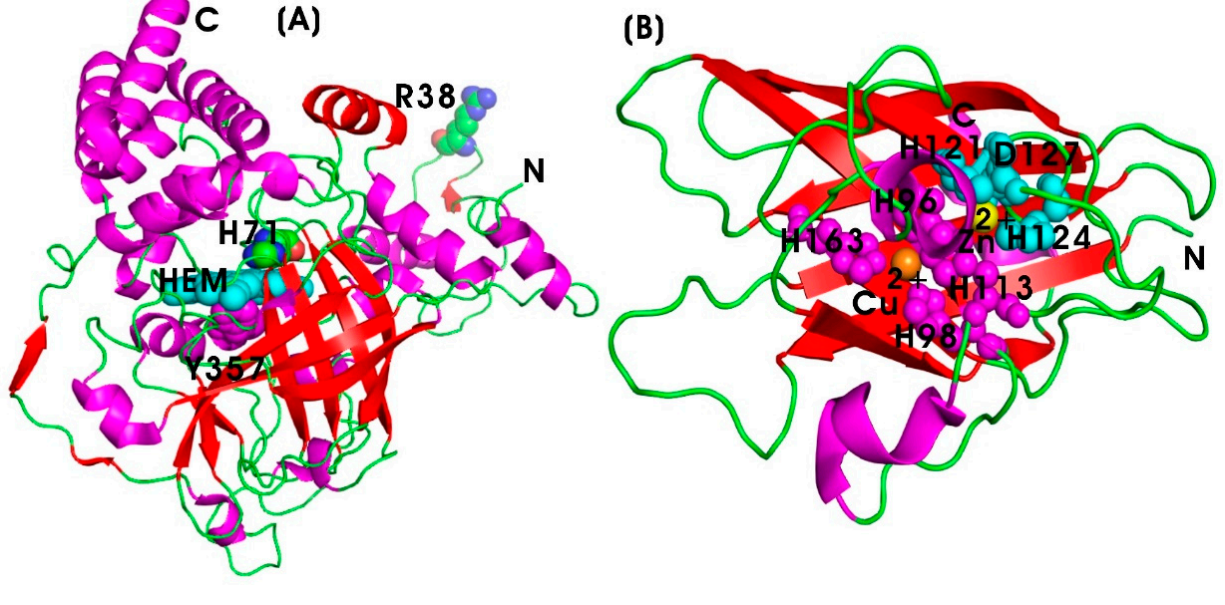
3D protein structures available in the protein data bank (PDB) for (**A**) catalase (PDB ID: 8EL9) and (**B**) superoxide dismutase (PDB ID: 2JLP), highlighting their active sites and metal ions.

Catalase (EC 1.11.1.6) is an antioxidant enzyme belonging to the oxidoreductase family, and it catalyzes the breakdown of hydrogen peroxide (H_2_O_2_), a reactive oxygen species (ROS), into water and oxygen. The primary function of catalase is to protect cells from oxidative damage caused by H_2_O_2_, which is toxic to cellular components [64]. Oxidative stress is a major contributor to chronic inflammation, and by neutralizing H_2_O_2_, catalase helps to mitigate oxidative damage and inflammatory responses. These effects are particularly relevant in autoimmune diseases such as rheumatoid arthritis, as well as conditions like myocardial infarction, cardiovascular disease (CVD), and neuroinflammation. ROS can activate transcription factors such as NF-κB, which mediate the expression of pro-inflammatory cytokines. Free radical scavenging enzymes like CAT inhibit the activation of pro-inflammatory signaling pathways, such as the NF-κB and MAPK pathways, thereby reducing cytokine production and inflammatory responses [65]. Recombinant human catalase has been utilized for both in vitro and in vivo therapeutic applications; however, its short half-life (~23 min in serum) limits its efficacy, necessitating specific targeting to the site of inflammation for optimal outcomes. The protein engineering of human CAT has improved the protease resistance in serum while maintaining its catalytic efficiency (7.3 × 10^6^ s^−1^ M^−1^). The active site of the CAT enzyme contains a heme group (protoporphyrin IX containing iron), which is essential for its catalytic function. The enzyme exists as a homo-tetramer and binds to NADPH, which stabilizes the enzyme and prevents inactivation by H_2_O_2_. The key amino acids involved in the catalytic process include histidine (H71), asparagine (R38), and tyrosine (Y357), facilitating the breakdown of H_2_O_2_ (Figure 4A). High levels of Reactive Oxygen Species (ROS) activate pro-inflammatory pathways, such as the NF-κB and MAPK signaling cascades, leading to the production of various pro-inflammatory mediators. In addition to this, ROS can damage cellular components and exacerbate inflammation [65,101]. Antioxidant enzymes play a critical role in alleviating inflammatory diseases by neutralizing ROS, thereby reducing oxidative stress in vivo [102,103,104]. Various metal oxide nanoparticles, including iron oxide (Fe_2_O_3_), zinc oxide (ZnO), ceria (CeO_2_), copper oxide (CuO), magnesium oxide (MgO), and titania (TiO_2_), along with metallic nanoparticles such as gold (Au), and copper (Cu), carbon-based nanoparticles (such as fullerene and graphene), and polymeric nanoparticles (such as polylactic-co-glycolic acid (PLGA) and chitosan) have demonstrated the free radical scavenging activities and modulation of antioxidant enzyme expression in biological systems [105,106,107,108,109,110,111,112].

Under oxidative stress conditions, nuclear factor erythroid 2-related factor 2 (Nrf2) functions as a critical transcription factor, translocating to the nucleus and binding to antioxidant response elements (ARE) in the promoters of antioxidant genes, thereby upregulating the expression of antioxidant enzymes [113]. Oxidative stress significantly contributes to the initiation and progression of inflammatory bowel disease (IBD), and the induction of CAT and superoxide dismutase (SOD) as free radical scavengers can mitigate the expression of pro-inflammatory mediators. In 2016, Zhang and colleagues synthesized an oxidation-responsive cyclodextrin material (OxbCD) incorporating the free radical scavenger Tempol (Tpl)-loaded OxbCD nanoparticles (Tpl/OxbCD NP). These nanoparticles were triggered by H_2_O_2_ for the controlled release of Tpl, effectively reducing ulcerative colitis and suppressing pro-inflammatory mediators, demonstrating their potential in IBD management [114]. Selenium nanoparticles (SeNPs) combined with p-coumaric acid have also exhibited therapeutic efficacy for inflammatory disorders such as acute gouty arthritis in RA rat models by increasing CAT and glutathione peroxidase (GPx1) mRNA levels while decreasing the mRNA levels of cyclooxygenase-2 (COX-2) [115].

Bioactive compounds derived from quail egg yolk oil (QEYO) increased CAT activity and total antioxidant activity while downregulating inflammatory genes Unpaired 2 (UPD2) and Tumor necrosis factor homolog (EIGER) in a Drosophila melanogaster model [116]. Furthermore, Aurozyme, a novel multi-scavenging nanozyme combining gold nanoparticles (AuNPs) and glycyrrhizin (GL) within a glycol chitosan coating layer, demonstrated a switch in the peroxidase-like activity of AuNPs to catalase-like activity. This switch enabled the scavenging of ROS/RNS and damage-associated molecular patterns (DAMPs), attenuating M1 macrophage polarization, and downregulating pro-inflammatory cascades in colitis models. Aurozyme also exhibited prolonged adhesion to inflammation sites that promote sustained anti-inflammatory activity, thus restoring intestinal function in colitis-challenged mice [117,118]. In 2019, Bang and colleagues reported that BST-104, a water extract of Lonicera japonica, exhibited gastroprotective effects in murine models of gastritis and peptic ulcers. This effect was attributed to increased CAT and SOD activities, reduced malondialdehyde (MDA) levels, and the suppression of pro-inflammatory cytokines via the downregulation of NF-κB expression [119].

Recently, a microalgae-based hybrid (SP@COS-CeO_2_) was developed using ceria nanoparticles (CeO_2_), which exhibit highly effective catalase-biomimetic activity, modified with positively charged chitosan oligosaccharides (COS). These COS-modified ceria nanoparticles were loaded onto the surface of the microalgae Spirulina platensis (SP), which contains abundant SOD, via electrostatic absorption, serving as a biocarrier. This biohybrid exhibited catalysis in the transformation of superoxide anion radicals into H_2_O_2_ by SP, which was then disproportionated into water and oxygen by CeO_2_ nanozymes, providing free-radical scavenging and anti-inflammatory activity in mouse models of ulcerative colitis and Crohn’s disease [120]. Since ROS plays a critical role in inflammatory diseases, scavenging ROS is important for facilitating anti-inflammatory cascades and regenerating damaged inflamed tissues. A pluronic-based nanocarrier loaded with dual antioxidant enzymes, SOD and CAT, has been developed as a nanoreactor system for the regeneration of inflammatory tissues. This system has facilitated a cascade reaction between SOD and CAT, converting superoxide anions into oxygen. Upon intravenous administration in vivo in IBD models, this SOD/CAT-loaded nanocarrier significantly enhanced tissue regeneration and alleviated inflammation, outperforming single enzyme (SOD or CAT)-loaded nanocarriers and free mixtures of both enzymes without the nanocarrier [121].

The therapeutic applications of CAT are often limited by its inherent sensitivity to physiological and environmental conditions. To overcome these limitations, various polymer-based conjugation strategies have been employed to enhance its stability and enzyme activity. One such approach involves the covalent conjugation of CAT to poly(acrylic acid) (PAA) via carbodiimide-mediated coupling chemistry, resulting in the formation of CAT-PAA conjugates. This formulation significantly improved key biophysical properties of CAT, including thermal stability (up to 85–90 °C), resistance to proteolytic degradation, and resistance to negatively charged inhibitors such as ascorbate, without change in their structure and catalytic activity [122]. These approaches highlight the potential of synthetic polymers as protective agents that enhance enzyme activity, offering a promising alternative to protein engineering for generating “Stable-on-the-Table” enzymes with extended shelf life and improved stability under harsh conditions. In another example, CAT was conjugated with methoxy-poly(ethylene glycol) (mPEG) to formulate CAT-PEG conjugates for topical applications. There was an inverse relationship between PEG molecular weight and specific enzyme activity; however, there was an improved long-term stability and retention of antioxidant activity. In particular, CAT-PEG20, where PEG had a molecular weight of 20 kDa, demonstrated complete inhibition of lipid peroxidation in the upper skin layers, making it suitable for therapeutic applications in vitiligo and other oxidative stress-associated dermal conditions [123].

#### 6.1.2. Superoxide Dismutase (SOD)

Endogenous oxidative stress (OS), characterized by the increased production of reactive oxygen metabolites (ROMs), inflammation, dysregulation of cellular redox homeostasis, mitochondrial dysfunction, and bioenergetic crisis, is recognized as a key factor contributing to the onset of various diseases and disorders [66]. Antioxidants play a crucial role in reducing oxidative stress and improving immune functions. Among them, antioxidant enzymes significantly influence the pathogenesis of various diseases. Superoxide dismutase (SOD) (EC 1.15.1.1), a critical antioxidant enzyme belonging to the oxidoreductase family, protects cells against oxidative stress by catalyzing the conversion of superoxide anions (O^2−^) into less toxic molecules. SOD is classified by its metal cofactors: copper (Cu^2+^)-zinc (Zn^2+^) SOD (Cu/Zn-SOD), including SOD1 (cytoplasmic and nucleus) and SOD3 (extracellular SOD; ecSOD), and is found in intracellular and extracellular spaces, respectively. Manganese (Mn^3+^) SOD (Mn-SOD or SOD2) is located in mitochondria, while iron SOD (Fe-SOD) is present in certain bacteria and plants [124]. In addition, SOD functions as a potential transcription factor, regulating the activity of various signaling pathways [125]. SOD converts superoxide anions into hydrogen peroxide (H_2_O_2_), which is subsequently converted into water by catalase or glutathione peroxidase. Figure 4B illustrates the human extracellular Cu/Zn-SOD3 structure at 1.7 Å resolution, revealing its homotetrameric structure. The metal-binding sites include Thr137 at the Cu-binding site and a shorter loop at the zinc-binding site. The N- and C-terminal end regions, essential for tetramerization and heparin-binding, respectively, are highly flexible. The metal-binding sites involve His96, His98, His113, and His163 acting as ligands to the Cu atom, while His113, His121, His124, and Asp127 coordinate the Zn site. The imidazole ring of His113 acts as a bridging ligand, forming coordination bonds with both Cu and Zn, thereby forming Cu–His113–Zn bridge [126].

Neutrophils are the primary immune cells recruited to sites of inflammation. Endothelial cells play a critical role in regulating neutrophil trafficking by controlling their adhesion and emigration from the bloodstream into the tissue site of inflammation under inflammatory conditions. Upon activation, neutrophils bind to the vascular endothelium and transmigrate into extravascular tissues, guided by chemokine gradients, participating in various inflammatory processes [127]. The activated neutrophils release ROS and proteases to eliminate pathogens but may also cause host tissue damage. Additionally, neutrophil-derived chemokines sustain inflammatory cell influx [128]. Dysregulated neutrophil activation is implicated in chronic inflammatory conditions such as rheumatoid arthritis, chronic obstructive pulmonary disease (COPD), and systemic lupus erythematosus (SLE) [129]. During oxidative stress, SOD functions as a natural endogenous cellular defense mechanism, breaking down superoxide anions into oxygen and hydrogen peroxide, which is further detoxified by glutathione peroxidase or catalase. Consequently, SOD has therapeutic potential in treating several neutrophil-mediated inflammatory diseases [130].

Furthermore, inflammation resolution requires the elimination of activated neutrophils via Fas-mediated apoptosis, which prevents the excessive release of pro-inflammatory cytokines and proteinases. SOD acts as a potential regulator of neutrophil-mediated inflammation by promoting neutrophil apoptosis [67]. The physiological balance between oxidants and antioxidants is crucial, as any imbalance contributes to the pathophysiology of several disorders. Superoxide anions stimulate endothelial cells and enhance neutrophil infiltration by producing chemotactic mediators such as leukotriene B4 (LTB4) and upregulating adhesion molecules like intracellular adhesion molecule (ICAM)-1. SOD-mediated inhibition of superoxide anions reduces neutrophil infiltration at inflammatory sites [131,132,133]. In 2020, Hwang and colleagues investigated the role of Cu/Zn-SOD (SOD1) in oxidative stress during colitis using a dextran sulfate sodium (DSS)-induced SOD-knockout (KO) mouse model of acute colitis. SOD1 deficiency led to severe oxidative stress, body weight loss, epithelial barrier disruption, and reduced antioxidant enzyme activity. Restoring SOD activity by the administration of *Bacillus amyloliquefaciens* SOD (BA SOD) to DSS-treated SOD-KO mice protected against colitis by inhibiting p38-MAPK/NF-kB signaling, which suggests the potential therapeutic use of BA SOD in colitis treatment [134]. Additionally, Lin and colleagues demonstrated that baculovirus-mediated SOD3 gene delivery in vascular smooth muscle cells (VSMCs) suppressed TNF-α-induced cell proliferation, migration, and inflammatory biomarkers expression. SOD3 overexpression also attenuated elevated levels of MMP-2 and MMP-9, further supporting its anti-inflammatory role [135].

Macrophages, as the essential component of the innate immune system, play a key role in all stages of inflammation, from initial immune response to resolution and tissue repair. Their tissue-specific nomenclature includes alveolar macrophages (lungs), Kupffer cells (liver), and microglia (brain) [136]. Based on the signals they receive, macrophages can differentiate into either M1 (pro-inflammatory) or M2 (anti-inflammatory) phenotypes [137]. In 2024, Kim and colleagues demonstrated that SOD inhibited the accumulation of ROS, enhanced the viability of hydrogen peroxide-treated nerve cells, reduced necrosis in nerve cells, and promoted the M1-to-M2 transition in macrophages, highlighting its neuroprotective and anti-inflammatory properties [138].

Initially, bovine SOD was used as systemic therapy to mitigate the adverse effects of ROS caused by radiation therapy in cancer patients and as local therapy for osteoarthritis pain. However, its use was discontinued due to severe anaphylactic reactions and fatalities. Subsequently, recombinant human SOD demonstrated immune tolerance and in vivo anti-inflammatory efficacy by significantly reducing inflammatory cytokine levels and alleviating inflammation-related symptoms in animal models. Despite these advances, genetically engineered recombinant human Cu/Zn SOD has a short half-life in serum (7–25 min), while human MnSOD, with a catalytic efficiency of 8 × 10^8^ s^−1^ M^−1^, also presents challenges in stability, necessitating innovative delivery strategies such as encapsulation or conjugation techniques to enhance stability and targeting to inflamed tissues. In 2005, Riedl and colleagues conducted clinical trials using liposomally encapsulated recombinant human SOD (IrhSOD) for the topical treatment of Peyronie’s disease. The approach significantly reduced pain and improved symptoms in 89% of patients without adverse effects [139]. Moreover, the targeted delivery of SOD to endothelial caveolae using biomolecules such as antibodies or cell-specific ligands demonstrated improved cell viability, reduced inflammation, and the decreased activation of pro-inflammatory mediators compared to non-targeted approaches [140]. These findings underscore the therapeutic potential of SOD in modulating oxidative stress, inflammatory responses, and immune regulation, paving the way for advanced enzyme-based anti-inflammatory treatments for inflammatory diseases.

Moreover, to enhance the therapeutic efficacy of anti-inflammatory enzymes, various chemical modifications have been explored to improve their pharmacokinetics, stability, and bioavailability. Among these strategies, PEGylation is well known and widely used, as it significantly increases the circulation half-life of enzymes, reduces their immunogenicity, and protects them from proteolytic degradation, thereby enhancing the overall pharmacokinetic profiles. However, despite these benefits, PEGylated enzymes have been reported to induce the formation of anti-PEG antibodies, which can lead to accelerated blood clearance (ABC) of PEG-conjugated proteins and liposomes, particularly those containing methoxyPEG (mPEG). Sherman et al. [141] demonstrated that PEG-protein conjugates synthesized using monofunctionally activated hydroxy-PEG (HO-PEG) exhibited lower antigenicity and immunogenicity compared to their mPEG-conjugated counterparts, suggesting that the chemical structure of PEG plays a crucial role in immunological responses. To overcome limitations associated with PEGylated, alternative polymer conjugates have been explored. For instance, SOD chemically modified with carboxymethyl cellulose (CMC) exhibited improved pharmacological properties, including a prolonged circulation half-life [142]. Similarly, the conjugation of Cu/Zn SOD with O-quaternary chitosan derivatives (O-HTCC) resulted in O-HTCC-SOD, which demonstrated enhanced therapeutic efficacy over native SOD. This conjugate showed a wider pH activity range, improved thermal and long-term storage stability, and an extended half-life in vivo [143]. Additional modifications, such as the amide linkage of SOD to O-carboxymethylchitin, resulted in glycosidated SOD, which exhibited a 2.4-fold increase in anti-inflammatory activity, higher resistance to hydrogen peroxide-mediated inactivation, and a significant increase in plasma half-life from 4.8 min to 69 h [144]. Similarly, glycosidation of bovine Cu/Zn-SOD with end-group aminated dextran enhanced anti-inflammatory activity by two-fold, exhibited increased oxidative resistance, and prolonged plasma half-life from 4 min to 3.2 h [145]. Lecithinized SOD, formed by covalently binding phosphatidylcholine palmitoyl to human recombinant SOD, improved both membrane affinity and half-life. This conjugate demonstrated a protective role against myocardial ischemia-reperfusion injuries in rats, attributed to increased bioavailability and efficient delivery to target tissues [146].

Despite these advancements, the limited permeability of antioxidant enzymes across biological barriers, such as the blood–brain barrier (BBB), remains a significant challenge in treating neuroinflammatory disorders. To address this, SOD1 was conjugated to amphiphilic poly(2-oxazoline) block copolymers, resulting in enhanced neuronal uptake compared to native or PEGylated SOD1. This conjugate also maintained high stability, both in serum and brain environments, offering potential for antioxidant therapy in superoxide-associated neurocardiovascular diseases, such as angiotensin II-dependent hypertension and heart failure [147]. Further, SOD1 conjugates with Pluronic block copolymers, specifically poly(ethylene oxide)-poly(propylene oxide)-poly(ethylene oxide) (e.g., SOD1-P85 and SOD1-L81), retained significant enzymatic activity and effectively penetrated neuronal membranes. These conjugates successfully inhibited angiotensin II-induced superoxide production within neurons, offering a promising approach to modulating intraneuronal redox signaling associated with cardiovascular dysfunctions [148]. Overall, these chemical modifications not only facilitate the targeted delivery of antioxidant enzymes to inflamed tissues but also enhance their pharmacokinetic behavior, reduce immunogenicity, and prolong systemic circulation for treating a wide spectrum of inflammatory diseases.

### 6.2. Hydrolases

Hydrolases are a class of enzymes that catalyze the hydrolysis of chemical bonds by adding water molecules into the substrate, leading to bond cleavage. The catalyzed reaction of hydrolase is as follows:

A–B + H_2_O → A–OH + B–H (2)

These enzymes cleave ester, glycosidic, peptide, amide, or phosphoester bonds and are essential for numerous biological processes, including digestion, metabolism, cell signaling, and tissue remodeling. Key subclasses of hydrolases include esterases, glycosidases, lipases, phosphodiesterases, phosphatases, peptidases or proteases, amidases, nucleases, and acid anhydride hydrolases [149].

#### 6.2.1. Serine and Cysteine Proteases

Proteases are crucial signaling molecules that regulate cellular functions by cleaving protease-activated receptors (PARs). These receptors are broadly expressed on various cell types, including platelets, epithelial cells, endothelial cells, and smooth muscle cells, as well as on innate and adaptive immune cells and cancer cells, and play significant roles in both physiological and pathological processes within the human body [150]. The activation of PARs by proteases mediates tissue repair and regeneration in response to injury. There are four major classes of PARs (PAR 1–4) belonging to the members of G-protein-coupled receptors (GPCRs). Among them, PAR1 and PAR2 are predominantly involved in inflammatory responses, including the recruitment of immune cells and vascular permeability [151]. Serine and cysteine proteases are enzymes involved in the breakdown of peptide bonds in proteins, and the primary difference is found in their active-site amino acid residues. Serine proteases contain a serine residue in their active site, whereas cysteine proteases contain a cysteine residue, which plays a critical role in their enzymatic activity [152].

##### Trypsin (TRYP) and Chymotrypsin (CHYMO)

Alkaline serine proteases, such as trypsin (TRYP) (EC 3.4.21.4) and chymotrypsin (CHYMO) (EC 3.4.21.1), typically degrade proteins and peptides to release free amino acids and small peptides. TRYP exhibits optimal activity at alkaline pH (7.5–8.5) at 37 °C, with calcium ions regulating their activity by stabilizing the enzyme structure, whereas CHYMO is optimally active at pH 7.5–8.0 [68]. Both these enzymes can activate various inflammatory mediators such as cytokines, chemokines, and PARs [153]. In 2023, Xiang and colleagues studied the in vitro and in vivo effects of low-dose TRYP (1–3000 nM) and PAR-2 agonist through PAR-2 activation on wound healing [154]. The results revealed that, similar to the natural repair mechanism mediated by mast cell tryptase, low-dose TRYP and synthesized PAR-2 agonists (2f-LIGRL-NH2 and SLIGRL-NH2) enhanced the migration, adhesion, and proliferation of fibroblasts and macrophages at wound sites and enhanced collagen deposition in skin wounds. Moreover, there was an upregulation of genes, including claudin-7, occludin, and IL-17A, associated with proliferation, migration, and focal adhesion, which contributed to wound healing. The oral combination of TRYP and CHYMO has been shown to effectively promote the healing of traumatic injuries. Its multifaceted properties include anti-inflammatory, anti-edematous, fibrinolytic, anti-infective, and analgesic effects, which improve recovery and help manage the inflammatory symptoms associated with tissue damage [69]. Given the involvement of multiple proteinases in inflammatory responses, a study by Abji and colleagues identified PAR2 and its activating proteinases, particularly tryptase-6, as significant mediators of inflammation in psoriatic arthritis (PsA). Elevated trypsin-like serine proteinase levels were observed in the synovial fluid (SF) of PsA and rheumatoid arthritis (RA) patients compared to those with osteoarthritis (OA), whereas chymotrypsin-like activity was higher in RA than in PsA. Tryptase-6 was identified as an active serine proteinase in synovial fluid that activated the calcium signaling pathway via PAR2, suggesting that targeting the proteinase-receptor axis could serve as a novel therapeutic approach for inflammatory diseases [70].

PARs are activated through extracellular N-terminus proteolytic cleavage by proteases such as MMPs, TRYP, CHYMO, or thrombin. This cleavage process exposes a tethered ligand (TL), an N-terminal sequence that binds to the receptor’s extracellular loop, inducing a conformational change and subsequent receptor activation. This process triggers downstream signaling cascades that mediate various biological responses. The immune response elicited depends on the specific proteases and PAR subtype involved [71]. Dysregulated protease activity has been implicated in immune dysfunction, visceral hypersensitivity, and mucosal barrier disruption in conditions such as irritable bowel syndrome (IBS) and inflammatory bowel disease (IBD) [155]. Targeting specific proteases on their receptors using protease modulators or inhibitors may provide a viable therapeutic strategy for reducing inflammation in inflammatory diseases. In addition, by targeting the protease-PAR interaction, therapeutic interventions can be developed to modulate these signaling pathways and enhance patient outcomes.

**Figure 5:**
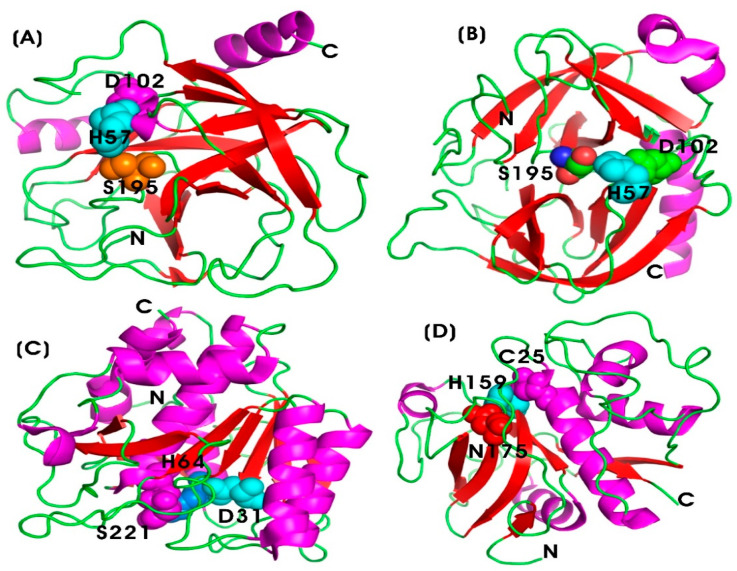
3D protein structures available in the protein data bank (PDB) for (**A**) trypsin (PDB ID: 1H4W), (**B**) chymotrypsin (PDB ID: 4CHA), (**C**) nattokinase (PDB ID: 4DWW), and (**D**) papain (PDB ID: 9PAP), highlighting their active sites.

In 2021, De bruyn and colleagues investigated the role of TRYP-like and elastase-like proteases in the cleavage and processing of bioactive peptides involved in colonic inflammation and pain. The results indicated that CHYMO-like proteases enhanced β-endorphin processing and disarmed the PAR3-based peptide during acute colitis. Moreover, thrombin activity was associated with the unmasking of the TL-sequence of the PAR1- and PAR4-based peptides. Thus, the study reveals that, in addition to TRYP and CHYMO, enkephalins and other proteases contribute to the complexity of protease interactions in IBD and IBS. Unraveling the specific roles of these proteases can facilitate their potential utilization as therapeutic targets for managing inflammatory conditions, thereby improving treatment strategies for patients affected with inflammatory diseases [156]. Figure 5A,B illustrates the structural characteristics of TRYP and CHYMO, which share a common catalytic triad composed of Ser195, His57, and Asp102, which are crucial for their proteolytic activity. Ser195 serves as the nucleophile responsible for the catalytic cleavage of peptide bonds; His57 acts as a base to activate serine residue at 195 (Ser195) by accepting a proton; and Asp102 stabilizes the positively charged histidine, thereby enhancing its catalytic function [157].

##### Nattokinase (NK)

Nattokinase (NK) is an alkaline serine protease enzyme (E.C. 3.4.21) composed of 275 amino acid residues, with a molecular weight of 27.7 kilodaltons (kDa). It lacks disulfide bonds and exhibits strong fibrinolytic activity. NK is produced through the fermentation of soybeans by the bacterium *Bacillus subtilis*, forming natto. It also activates other fibrinolytic enzymes, such as pro-urokinase and tissue plasminogen activator [72,73]. Figure 5C illustrates the catalytic triad of nattokinase, comprising Ser221, His64, and Asp31, which are crucial for its proteolytic activity [158]. NK functions as a fibrinolytic enzyme, facilitating the breakdown of fibrin in blood clots, which is crucial for maintaining proper circulation and preventing thrombosis. Additionally, it exhibits anti-inflammatory properties [159]. Furthermore, NK is recognized for its excellent biological activities, including anti-tumor, antiplatelet, lipid-lowering, antihypertensive, and neuroprotective effects [74,75,76]. Notably, it has been correlated with antioxidant activity due to its high levels of menaquinones-7, which contribute to neuroprotection against neurodegenerative diseases [160]. The anti-thrombotic mechanism of NK is attributed to its ability to disrupt the pathological loop between inflammation, oxidative stress, and coagulation [161]. In 2023, Liang and colleagues showed that genetically engineered recombinant probiotic *Escherichia coli* Nissle 1917 (EcN) expressing nattokinase (EcNnatto) enhanced therapeutic efficacy in colitis models in mice. This improvement was achieved through the regulation of gut flora and the maintenance of intestinal barrier integrity, leading to increased colon length and downregulation of pro-inflammatory cytokines such as IL-6 and TNF-α. Additionally, NK expression in EcN mitigated dextran sulfate sodium (DSS)-induced epithelial damage, restoring mucosal integrity by upregulating tight junction proteins, including Zonula Occludens-1 (ZO-1) and occluding. It also increased the expression of the intestinal stem cell marker Lgr5 (leucine-rich repeat-containing G-protein coupled receptor 5), which contributed to intestinal inflammation treatment [162].

NK has also shown potential in the prevention and treatment of cardiovascular diseases. It exhibits anti-angiogenic effects against retinal neovascularization via modulation of the Nrf2/HO-1 (nuclear factor erythroid 2-related factor 2/heme oxygenase-1) pathway, reducing glial activation and neuroinflammation. This suggests its potential as a therapeutic strategy for retinal neovascularization [76]. Moreover, NK has been proposed as an adjuvant therapeutic strategy for various non-communicable diseases (NCDs) by mitigating molecular pathways involved in inflammation and oxidative stress [163]. Interestingly, Wu and colleagues demonstrated the efficacy of NK in preventing xylene-induced ear edema and protecting against lipopolysaccharide (LPS)-induced acute kidney injury (AKI) by inhibiting inflammatory processes and oxidative stress in mice. In vivo studies using RAW264.7 macrophages showed that NK suppresses inflammation by inhibiting TLR4 and NOX2 activation, leading to decreased ROS production, the downregulation of MAPK activation, and reduced NF-κB translocation. This cascade results in decreased expression of pro-inflammatory cytokines such as TNF-α, IL-6, NO, and PAI-1 in activated macrophages [161]. Additionally, NK has been found to prevent hyperglycemia-induced inflammation in streptozotocin (STZ)-induced type 2 diabetes mellitus by inhibiting the formation of advanced glycation end products (AGEs) and resulting in decreased glycogen deposition in renal tubules, thereby mitigating diabetic nephropathy progression in diabetic patients [164].

##### Bromelain (BROM)

Bromelain (BROM) (EC 3.4.22.33) is a complex plant enzyme extract derived from the fruits and stems of *Ananas comosus* (pineapple), a member of the Bromeliaceae family. It consists of enzymatic and non-enzymatic components, including antioxidants, carotenoids, flavonoids, and organic acids (ascorbic acid, malic acid, citric acid), which are known for their cytoprotective properties against free radical-induced damage [77]. BROM belongs to the cysteine protease family (peptidase C1 family, also known as the papain-like family) and contains proteolytic enzymes with endopeptidase activity, including comosain, ananain, glucosidase, cellulase, peroxidases, and phosphatases [16]. In recent years, extensive research has been conducted to explore the therapeutic potential of bromelain in various disorders and diseases due to its anti-inflammatory, immunomodulatory, anticoagulant, anti-neoplastic, and antimicrobial properties [78,79,80,81]. Its anti-inflammatory efficacy is comparable to those of non-steroidal anti-inflammatory drugs (NSAIDs), but with fewer side effects.

The anti-inflammatory and analgesic effects of BROM have garnered particular interest in the treatment of osteoarthritis and rheumatoid arthritis (RA). Molecular mechanisms underlying these effects involve the modulation of various signaling pathways associated with inflammation, pain, and tissue damage. In osteoarthritis, BROM reduces symptoms by inhibiting the expression of pro-inflammatory cytokines, including interleukin-1β (IL-1β), IL-6, monocyte chemoattractant protein-1 (MCP-1), and TNF-α, which contribute to inflammation, cartilage degradation, and joint tissue damage [165,166,167]. Furthermore, BROM inhibits cyclooxygenase-2 (COX-2), a key enzyme in the synthesis of inflammatory prostaglandins through the arachidonic pathway, which plays a role in osteoarthritis-associated inflammation [168,169]. Both in vitro and in vivo studies have demonstrated BROM’s ability to inhibit matrix metalloproteinases (MMPs), particularly MMP-3 (stromelysin-1) and MMP-13 (collagenase-3), which degrade extracellular matrix (ECM) proteins such as collagen and aggrecan, contributing to cartilage breakdown in osteoarthritis. By inhibiting these MMPs, BROM helps preserve cartilage integrity [170,171].

A clinical trial evaluating the efficacy of BROM in patients with moderate-to-severe RA or osteoarthritis revealed that patients administered a combination of BROM, rutin, and trypsin exhibited reduced inflammation and pain. In contrast, patients treated with NSAID diclofenac developed peptic ulcers. These anti-inflammatory properties of BROM are attributed to various mechanisms, including decreased plasma fibrinogen levels, reduced bradykinin activity, increased serum fibrinolytic activity, decreased levels of prostaglandin E2 (PGE2) and thromboxane A2 (TXA2), and the modulation of adhesion molecules, such as intercellular adhesion molecule -1 (ICAM-1), vascular cell adhesion molecule-1 (VCAM-1), E-, P- and L-selectin (endothelial-, platelet- and leukocyte-selectin), CD44, and integrins (α4β1 and αLβ2) on immune cell surfaces involved in the pathogenesis of RA [172,173,174,175,176].

An animal model study demonstrated that the administration of varying doses of methanolic extract from pineapple fruit peel reduced paw swelling and inflammatory biomarkers (CAT and GPx in the liver, CRP, and PGE2) in rats with Complete Freund’s Adjuvant (CFA)-induced RA [177]. Similarly, the oral administration of BROM exhibited significant protective effects against the symptoms of intestinal inflammation by effectively modulating oxidative stress biomarkers and pro-inflammatory cytokines in indomethacin-induced intestinal inflammation in rats [178]. These findings suggest that BROM could be a viable therapeutic alternative for inflammatory conditions, offering advantages over conventional anti-inflammatory treatments due to its multifaceted biological effects and favorable safety profile.

##### Papain (PAP)

Papain (PAP) (EC 3.4.22.2) is an endolytic plant cysteine protease derived from the latex of the raw papaya fruit (*Carica papaya* L.), classified within the thiol proteinase family. It consists of a single polypeptide chain composed of 212 amino acids with a molecular weight of 23.3 kDa. The enzyme is structurally stabilized by three disulfide bridges and features a three-dimensional configuration with two distinct domains separated by a cleft containing the active site. This active site contains a catalytic dyad that is analogous to the catalytic triad found in serine peptidases like chymotrypsin [82,179,180]. Figure 5D illustrates the catalytic residues essential for its proteolytic function. The catalytic triad―Cys25, His159, and Asn175―is crucial for its catalytic activity. Cys25 serves as a nucleophile, facilitating peptide bond hydrolysis by attacking the substrate’s carbonyl carbon. His159 functions as a general acid/base, stabilizing the thiolate form of Cys25 and promoting proton transfer during catalysis. Asn175 provides additional stability to the catalytic histidine through hydrogen bonding and electrostatic interactions. Furthermore, Gln19 and the backbone amide of Cys25 contribute to the formation of an oxyanion hole, which stabilizes reaction intermediates during proteolysis [83]. PAP exhibits remarkable stability under elevated temperatures, denaturing agents (such as 8 M urea), and organic solvents (including 70% ethanol). Its optimal enzymatic activity occurs within a pH range of 3.0 to 9.0 [181,182].

The enzyme has broad substrate specificity, hydrolyzing various peptide bonds, making it valuable for pharmaceutical and biomedical applications [83]. It is frequently incorporated into topical formulations and dietary supplements for managing inflammation, arthritis, and soft tissue injuries [82]. The therapeutic potential of PAP in inflammatory conditions has been well-documented. In 1985, Mansfield and colleagues reported its analgesic and anti-inflammatory efficacy in alleviating symptoms of acute allergic sinusitis, including headaches and toothaches, without adverse effects. Additionally, oral administration of PAP has been shown to suppress inflammatory mediators, enhance the expression of antioxidant enzymes, and downregulate key signaling pathways, including mitogen-activated protein kinases (MAPKs) and the signal transducer and activator of transcription (STAT), which play critical roles in atopic dermatitis-like skin inflammation [84].

Recent studies highlight PAP’s potential in treating atopic dermatitis exacerbated by exposure to house dust mites (Dermatophagoides farina body, Dfb) in an NC/Nga atopic dermatitis mice model and human HaCaT keratinocytes in vitro [183]. The enzyme also influences monocyte activation, a crucial factor in the early pathogenesis of various inflammatory diseases. Monocyte-platelet aggregates (MPAs) play a significant role in mediating inflammatory responses. In 2018, Fei and colleagues demonstrated that PAP mitigates MPA formation-mediated monocyte activation by downregulating COX-1 expression through the modulation of the MAPKs and PI3K/Akt signaling pathways [184]. Furthermore, Jiang and colleagues reported that PAP inhibits MPA-mediated inflammatory responses by suppressing the NF-κB signaling pathway in activated monocytes, leading to the upregulation of microRNA-146a (miRNA-146a) transcription, which in turn downregulates pro-inflammatory cytokines and reduces inflammation. Additionally, PAP suppresses both COX-2 mRNA and protein expression, as well as MCP-1 levels in activated monocytes, further underscoring its potential as an anti-inflammatory agent [185].

#### 6.2.2. Metalloprotease

Metalloproteases, also known as metalloproteinases (MMPs), constitute a diverse family of zinc- or cobalt-dependent endopeptidases that play a pivotal role in ECM remodeling, inflammation modulation, and immune response regulation. These enzymes are characterized by a highly conserved catalytic domain, which contains zinc- or cobalt-binding motifs essential for their proteolytic activity. MMPs contribute to a range of physiological and pathological processes, including wound healing, tissue remodeling, and inflammatory response regulation [186]. Understanding the intricate relationship between metalloprotease-mediated ECM degradation and inflammation suppression is essential for developing effective enzyme-based anti-inflammatory therapeutics.

##### Serratiopeptidase (SERR)

**Figure 6:**
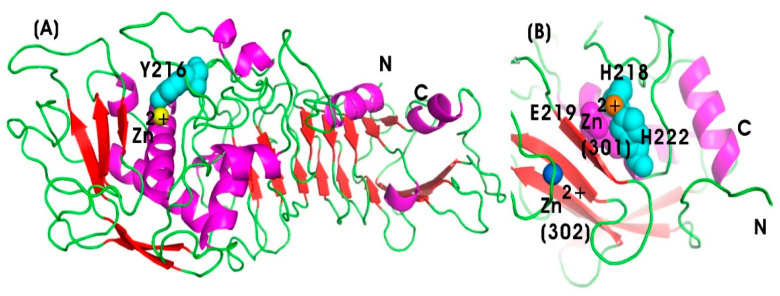
3D protein structures available in the protein data bank (PDB) for (**A**) serratiopeptidase (PDB ID: 1SAT) and (**B**) collagenase (PDB ID: 1CGE), highlighting their active sites and metal ions.

Serratiopeptidase (SERR) (EC 3.4.24.40), also known as serrapeptase or serralysin, is a bacterial metalloprotease hydrolase enzyme belonging to the peptidase M10 family of matrix metalloproteinases (MMPs). It is derived from the rod-shaped, Gram-negative bacterium *Serratia* sp. E15 (S. marcescens ATCC 21074) [187,188]. It was first introduced by Japanese researchers in the 1970s as the first natural anti-inflammatory drug, serving as a natural alternative to synthetic nonsteroidal anti-inflammatory drugs (NSAIDs) [85,86]. SERR is a 50-kDa metalloprotease enzyme with zinc as a cofactor, with a molecular weight of 45–60 kDa. It belongs to the serralysin group, consisting of 470 amino acids, and it exhibits optimal proteolytic activity at pH 9.0 and 40 °C [189]. Structurally, SERR shares similarities with the alkaline protease from Pseudomonas aeruginosa, particularly in its calcium-binding motif. The N-terminal proteolytic domain exhibits the typical zinc-dependent metalloprotease structural motif of the metzincin fold. However, the active site of serratiopeptidase includes Tyr216, a Zn ligand, along with loops 70–77 and 122–132, which form the active site cleft [190] (Figure 6A).

Cyclooxygenases (COX-1 and COX-2) are key enzymes responsible for the breakdown of arachidonic acid via the cyclooxygenase pathway, leading to the production of inflammatory mediators such as interleukins and prostaglandins [87,188]. NSAIDs exert anti-inflammatory effects by strongly interacting with COX enzymes and inhibiting the synthesis of these mediators. Similarly, SERR has been shown to bind to COX enzymes and suppress the release of interleukins, prostaglandins, and thromboxanes, although it does not interfere with the lipoxygenase (LOX) pathway [86,87]. SERR demonstrates superior anti-inflammatory activity both independently and in combination with other drugs [88]. Preclinical studies have reported that SERR-treated mice exhibit significantly lower CRP levels and decreased myeloperoxidase activity, an inflammatory enzyme marker. In a 2010 study, Jadav and colleagues reported that orally ingested SERR was found to have anti-inflammatory efficacy comparable to diclofenac sodium in both chronic and acute inflammation models [191]. In addition to its anti-inflammatory properties, SERR also possesses antibacterial and antibiofilm activities. When combined with vancomycin and rifampicin, SERR has been shown to inhibit biofilm formation by methicillin-resistant Staphylococcus aureus (MRSA) [192]. Additionally, SERR exhibits mucolytic activity, as demonstrated in studies where it was used in combination with the enzyme seaprose. It also displays fibrinolytic properties, making it a valuable component in fibrinolytic therapy for clot dissolution [193]. Recent studies have further highlighted its potential cytotoxic activity against colon cancer cell lines (Caco-2), suggesting potential anticancer applications [194,195]. These multi-faceted properties of SERR position it as a promising candidate for enzyme-based therapeutics targeting inflammation, bacterial infections, and cancer.

##### Collagenase (COLL)

Collagenase (COLL) (EC 3.4.24.3) is a type of metalloproteolytic enzyme responsible for the breakdown of collagen, the principal structural protein in the ECM of connective tissues, including skin, tendons, ligaments, and cartilage [196]. Matrix metalloproteinases (MMPs) are activated by calcium and are dependent on zinc or cobalt for enzymatic activity. COLL plays a vital role in tissue remodeling, wound healing, and other biological processes associated with connective tissue degradation [89]. These functions are critical for both inflammatory and anti-inflammatory responses. COLL is categorized within the MMPs family, which facilitates ECM degradation. Specifically, MMP-1 and MMP-8, known as interstitial and neutrophil collagenases, respectively, contribute to collagen degradation and the resolution of inflammation by promoting tissue repair [197]. Figure 6B illustrates the protein structure of the 19-kDa human fibroblast collagenase, highlighting the catalytic domain, which is spherical with an active site cleft containing a ligated catalytic zinc ion [198]. The histidine residues at positions 218 and 222 coordinate the catalytic zinc ion, while glutamate (Glu219) functions as a general base, facilitating nucleophilic attack on the substrate. The C-terminal domain is involved in substrate binding and in positioning the catalytic domain for enzymatic cleavage [199]. The zinc ion (Zn^2+^) stabilizes the transition state and activates water molecules required for peptide bond cleavage. Zinc at the 301 position is essential for enzymatic activity, while zinc at 302 and calcium at 303 plays a crucial role in maintaining the structural integrity of human fibroblast collagenase by stabilizing the extended loop (residues 165–181) between β strands [200].

COLL exhibits both pro-inflammatory and anti-inflammatory effects [90]. Controlled collagenase activity is essential for anti-inflammatory responses and mitigating chronic inflammation, whereas dysregulated activity exacerbates inflammatory conditions, such as rheumatoid arthritis [91]. Additionally, COLL aids in wound healing by reducing inflammation through the removal of collagen that perpetuates the inflammatory process. The enzyme is also involved in breaking down excess collagen associated with scarring or fibrosis, thereby reducing fibrosis in anti-fibrotic therapies [92]. Therapeutically, COLL is utilized in the treatment of Dupuytren’s contracture and Peyronie’s disease, where it effectively degrades abnormal collagen buildup, reducing tissue stiffness and associated inflammatory conditions [93].

Bacterial collagenases serve as key virulent factors due to their ability to degrade the collagen of the ECM. Clostridium histolyticum-derived collagenase is used in debridement, a process that removes damaged and necrotic tissues to promote active healing. This enzyme releases collagen-derived peptides that enhance macrophage chemotaxis and stimulate cytokine release, thereby accelerating wound healing. Santyl ointment, containing collagenase A from clostridial collagenase along with other generic proteases, is a well-established enzymatic debridement treatment [201]. Clostridial collagenase (CC) has been reported to exhibit anti-inflammatory effects both in vitro and in vivo [202]. The digestion of collagen by CC produces peptides that elicit cellular responses consistent with in vivo wound healing. In 2015, Galperin and colleagues demonstrated that the debridement of mildly inflamed diabetic foot ulcers with CC ointment (CCO) led to inflammation resolution. In addition, in vitro studies indicated that collagenase-digested collagens suppressed the production of lipopolysaccharides (LPS)-induced pro-inflammatory cytokines (TNF-α and IL-6) in differentiated THP-1 monocytes. This finding suggests a decrease in inflammation and an enhancement in the wound-healing process [202]. In 2021, Frederick and colleagues reported the use of Clostridium collagenase for debridement, which modulated cellular responses to facilitate an anti-inflammatory microenvironment conducive to a coordinated healing response. In a classic burn comb model in pigs, collagenase treatment prevented dermal collagen destruction and reduced early markers of necrosis (High Mobility Group Box 1; HMGB1) and apoptosis (cleaved caspase-3; CC3a) in burn injuries. This treatment also increased cellular infiltration and protected the tissue from burn wound conversion. Additionally, it significantly increased macrophage polarization towards Major Histocompatibility Complex Class II (MHC II) and supported vascular network maintenance, indicating the establishment of a pro-resolving macrophage environment [203].

In 2010, the Food and Drug Administration (FDA) approved the use of collagenase derived from Clostridium histolyticum (CCH), marketed as Xiaflex (Auxilium Pharmaceuticals, Malvern, PA, USA), for the safe, effective, and minimally invasive treatment of the fibroproliferative disorder Dupuytren contracture (DC). Mickelson and colleagues reported the clinical correction of DC within seven days following CCH injection [204]. NSAIDs such as celecoxib are often used as adjuvant therapy to enhance outcomes in DC patients with high fibrosis diathesis after treatment with CCH [205,206]. In patients with lumbar disc herniation, low-temperature plasma ablation combined with COLL injection (LTC) has shown superior efficacy compared to low-temperature plasma ablation alone, resulting in faster recovery and reduced immune stress responses [207]. Additionally, COLL has been produced by Vibrio alginolyticus and Bacillus cereus [208,209]. These findings highlight the diverse therapeutic potential of collagenase in managing inflammatory diseases, reducing fibrosis, and promoting wound healing, making it a promising enzyme-based anti-inflammatory therapeutic.

#### 6.2.3. Glycoside Hydrolase

Glycoside hydrolases, also referred to as endolytic glycosidases or glycosyl hydrolases family 56, are enzymes that catalyze the hydrolysis of glycosidic bonds between saccharide moieties of carbohydrates. These enzymes are widely distributed across various biological systems and play critical roles in cellular metabolism, the degradation of polysaccharides, and the regulation of ECM components. Their enzymatic activity is crucial for maintaining tissue homeostasis and modulating inflammatory responses.

##### Hyaluronidase (HYAL)

**Figure 7:**
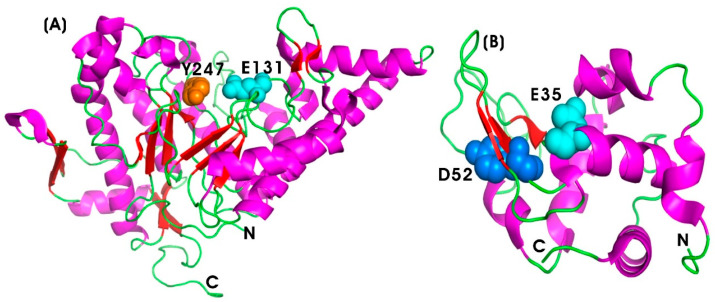
3D protein structures available in the protein data bank (PDB) for (**A**) hyaluronidase (PDB ID: 2PE4) and (**B**) lysozyme (PDB ID: 1I1z), highlighting their active sites.

Mammalian hyaluronidase (HYAL) (EC 3.2.1.35), classified as endo-β-N-acetyl-hexosaminidases, is a specific type of glycoside hydrolase belonging to family 56. It degrades hyaluronic acid (HA), a glycosaminoglycan (GAG) heteropolysaccharide and a crucial component of the ECM in human tissues [210]. This enzyme facilitates the cleavage of the β-1,4 glycosidic covalent bond between N-acetyl-D-glucosamine (GlcNAc or NAG) and D-glucuronic acid (GlcA or GA), leading to the depolymerization of hyaluronic acid and the formation of smaller oligosaccharides [211]. Human Hyal-1 and Hyal-2 are expressed in most tissues and are responsible for the catabolism of intracellular and extracellular hyaluronan, respectively [212]. Figure 7A illustrates the 3D protein structure of human hyaluronidase-1, a 435 amino acid residue protein with optimal enzymatic activity at an acidic pH range of 3.0–4.5. Human hyaluronidase-1 (HYAL1), an isoform of HYAL, consists of an N-terminal catalytic domain (Phe22–Thr352) and a smaller C-terminal domain (Ser353–Trp435), with active site residues located at Glu131 and Tyr247. Glu131 acts as a proton donor for the hydroxyl leaving group, whereas Tyr247 stabilizes the catalytic nucleophile, which forms a resonance intermediate with N-acetylglucosamine on the substrate [213,214].

Intriguingly, the molecular weight of HA is crucial for its diverse biological functions [210,215]. High-molecular-weight hyaluronic acid (HMW-HA, >1000 kDa) typically serves as a space-filling molecule that maintains tissue hydration and homeostasis. Furthermore, under inflammatory conditions, HMW-HA has been found to mitigate inflammatory responses due to its anti-inflammatory, anti-angiogenic, antioxidant, and immunosuppressive properties, promoting tissue regeneration and wound healing [94,95,96]. HMW-HA is degraded by hyaluronidases, and HYAL-1 and -2 are responsible for the majority of LMW-HA production in somatic tissues. In 2013, Tian and colleagues showed that HMW-HA possesses inherent anti-aging and anti-cancer properties [97]. In contrast, low-molecular-weight HA (LMW-HA; 150–350 kDa) has been associated with pro-inflammatory activities, leading to increased levels of macrophage inflammatory protein-1α and monocyte chemotactic protein-1, which exacerbate inflammation [98].

Although HYAL is not explicitly categorized as an anti-inflammatory enzyme, it contributes to inflammation resolution through the modulation of ECM components, edema reduction, and the promotion of tissue healing, thereby offering indirect anti-inflammatory effects through the regulation of inflammation. For instance, HYAL has been shown to reduce cerebral edema following traumatic brain injury by degrading hyaluronan in mouse models [216]. Furthermore, HYAL accelerates wound healing by enhancing the migration and proliferation of fibroblasts at low concentrations (0.1 U HYAL) in vitro. Additionally, HYAL promotes re-epithelialization in adult Wistar rats generated with full-thickness excisional wounds. Histological and biochemical analyses of these wounds have indicated that HYAL treatment enhanced granulation tissue formation, reduced edema, and regulated the inflammatory response by modulating the release of pro- and anti-inflammatory cytokines, growth factors, and eicosanoid mediators. Moreover, HYAL was found to upregulate the expression of peroxisome proliferator-activated receptors (PPAR-γ and PPAR-β/δ), collagen content, and angiogenesis, all of which contribute to efficient wound healing [217]. HYAL facilitates immune cell infiltration, particularly of neutrophils and macrophages, by degrading ECM-bound HA at injury sites, thereby expediting inflammation resolution [218]. Furthermore, HYAL exerts multiple effects on HA metabolism and skin structural cells, including HA synthesis in a dose-dependent manner, with the lowest effective concentration of HYAL (0.015 U/mL) [219].

##### Lysozyme (LYS)

Lysozyme (LYS) (EC 3.2.1.17), also known as mucopeptide N-acetyl-muramyl hydrolase, belongs to the glycoside hydrolase enzyme class. It catalyzes the hydrolysis of the β-1,4-glycosidic bond between N-acetylglucosamine and N-acetylmuramic acid in the peptidoglycan cell walls of Gram-positive bacteria, resulting in bacteriolysis. LYS is predominantly found in egg white, saliva, human tears, and mucous secretions. It plays a crucial role in the innate immune system by reducing tissue swelling associated with bacterial infections and modulating inflammatory responses. LYS decreases the release of pro-inflammatory cytokines such as interleukins (IL-1β, IL-6) and TNF-α by interacting with macrophages and neutrophils, thereby promoting tissue repair. Transcriptome profiling of U937 monocyte cells treated with LYS has demonstrated that LYS induces transcriptional modulation of genes within the TNF-α/IL-1β pathway, further confirming its anti-inflammatory activity [99]. Figure 7B illustrates the 3D protein structure of human LYS. The active site of the enzyme consists of Asp52 and Glu35, which are crucial for its catalytic function. Glu35 acts as a proton donor, facilitating the cleavage of the glycosidic bond by donating a proton to the leaving oxygen, generating an oxocarbenium-like transition state. Asp52 stabilizes this positively charged oxocarbenium ion high-energy intermediate, either through electrostatic interactions or by forming a transient covalent bond during glycosidic bond cleavage [220].

The potential of LYS as a therapeutic agent has been explored through innovative biomaterials. The fabrication of Cu^2+^-loaded phase-transitioned LYS nanofilm on bacterial cellulose (BC/PTL/Cu) composite demonstrated antibacterial and wound-healing properties in Sprague Dawley (SD) rats. This composite enhanced epithelial and granulation tissue regeneration by promoting collagen deposition, reducing IL-1β and TNF-α expression, and stimulating pro-angiogenic responses in vivo [221]. Tagashira and colleagues demonstrated the anti-inflammatory properties of LYS by suppressing lipopolysaccharide (LPS)-induced inflammatory responses in both in vitro and in vivo inflammation models using mouse macrophages (RAW 264.7 cells) and mouse peritoneal macrophages, respectively. The oral administration of high-dose LYS (2250 mg/kg body weight/day) in LPS-induced inflammation models in mice significantly reduced serum levels of IL-6 and TNF-α, as well as the downregulation of IL-1β and IL-12 expression [222]. Additionally, high-dose LYS administration resulted in reduced IL-6 levels in the liver. The suppression of IL-6 gene expression was also observed in the spleen for both the middle-dose group (450 mg/kg body weight/day) and the high-dose group [223]. These results suggest that LYS alleviates LPS-induced inflammation both in vitro and in vivo through the suppression of inflammatory cytokines. While NSAIDs are widely used and effective against inflammatory diseases, their use is associated with various adverse effects. Therefore, the identification and evaluation of anti-inflammatory potentials of different natural and novel compounds are urgently needed as safer alternatives. LYS has proven to be a natural source of anti-inflammatory agents and an alternative to the use of NSAIDs. Carrillo and colleagues (2016) evaluated the anti-inflammatory properties of native and modified hen egg white LYS using a carrageenan-induced paw edema model in mice. Their results suggested that LYS denatured by heat treatment and dithiothreitol (DTT) exhibited significantly higher anti-inflammatory activity, with a 39.47% and 42.10% inhibition of paw edema, respectively, when administered orally at a dose of 30 mg/kg of animal weight. Furthermore, the study suggested that the anti-inflammatory activity of denatured LYS was higher than that of hydrolyzed or native LYS at the same dosage, as well as that of glucocorticoid dexamethasone. This enhanced activity of denatured LYS was attributed to the unfolding of LYS, which exposes its hydrophobic core and positively charged amino acids. These structural changes may have facilitated interactions with and neutralization of the inflammatory mediators involved in paw edema formation [224].

Further research by Ibrahim et al. [225] identified five antimicrobial peptide motifs within the N-terminal region of LYS that are generated by pepsin activity, which exhibited potent anti-inflammatory properties. These five peptides include helix 1 (H1), helix 2 (H2), H1-loop-H2 (HLH), and H2 extended with either two β-strands (H2-S12) or three β-strands (H2-S13). Each of these peptides has been shown to dose-dependently reduce the expression of pro-inflammatory cytokines with varying degrees. Among the five peptides, the HLH peptide, along with its individual helices (H1 and H2), displayed the most potent anti-inflammatory activity in LPS- or interferon-gamma (IFN-γ)-stimulated mouse RAW264.7 macrophage cells. Molecular docking simulations and receptor binding assays revealed that the H1 peptide of LYS specifically binds to TLR4 on macrophages, suppressing inflammatory signaling. Thus, these LYS-derived therapeutic peptides provide insights into managing inflammatory disorders [225]. LYS has also been explored as an adjunct therapy for chronic obstructive pulmonary disease (COPD). Fukuchi and colleagues (2016) evaluated the long-term effects of orally administrated LYS (270 mg/day) in patients with moderate-to-severe COPD with one or more episodes of COPD exacerbation, alongside standard therapies such as bronchodilators. Over a 52-week period, LYS significantly reduced COPD exacerbation rates and improved forced expiratory volume in one second (FEV1) and COPD assessment test scores, particularly in airway-dominant phenotypes compared to the placebo group [226].

Recombinant technology has facilitated the development of anti-inflammatory LYS variants. The invertebrate-type LYS gene from *Scylla paramamosain* (mud crab), termed Splys-I, was cloned and overexpressed in the recombinant *Escherichia coli* (*E. coli*) Rossetta (DE3) strain. Purified Splys-I inhibited the phosphorylation of IκBα and NF-κB (P65), leading to decreased secretion of pro-inflammatory cytokines [227]. In poultry, microbial LYS supplementation (e.g., Lysonir) has demonstrated effectiveness as an antibiotic alternative, reducing the levels of IL-1β and IL-8 (chemokine (C–X–C motif) ligand 8, CXCL8), while enhancing intestinal integrity, feed efficiency, and anti-inflammatory and immune responses in broiler chickens [228].

Systemic inflammatory responses remain a leading cause of mortality in hospitals worldwide. High mobility group box 1 (HMGB1) is a nonhistone chromosomal protein, characterized by high electrophoretic mobility, which exhibits pro-inflammatory properties when released by cells. HMGB1 is implicated as a key extracellular mediator in severe vascular inflammatory diseases and sepsis. It also stimulates various receptors, contributing to the pathogenesis of several human diseases, making it an important therapeutic target for the clinical management of systemic inflammatory conditions [229]. Lee et al. (2015) [230] demonstrated the anti-septic effects of LYS. LYS inhibits HMGB1-mediated inflammatory responses in human umbilical vein endothelial cells (HUVECs) in vitro, as well as in septic C57BL/6 male mice in vivo. LYS reduced LPS- and cecal ligation and puncture (CLP)-induced HMGB1 release while downregulating its receptors, including TLR2, TLR4, and the receptors for advanced glycation end products (RAGE). Furthermore, LYS improved vascular integrity by inhibiting the expression of cell adhesion molecules (CAMs) such as the vascular cell adhesion molecule (VCAM), the intercellular adhesion molecule (ICAM), and E-selectin. In vivo studies further confirmed a reduction in HMGB1-induced mortality in mouse models of sepsis. Thus, LYS may be effectively employed in the treatment of septic shock and the management of severe systemic inflammatory responses [230].

## 7. Anti-Inflammatory Engineered Enzymes

Natural enzymes, although effective in various therapeutic applications, often exhibit limitations, such as a short half-life under physiological conditions, immunogenicity, off-target effects, and instability in therapeutic environments. To overcome these challenges, enzymes are modified using advanced protein engineering techniques such as rational design, directed evolution, site-directed mutagenesis, fusion protein technology, and chemical conjugation [39,41]. These strategies are designed to enhance substrate specificity, improve structural and thermal stability, reduce immunogenicity and toxicity, and increase bioavailability. Thus, engineered enzymes exhibit improved pharmacokinetic properties, including an extended half-life, enhanced stability, and minimized immune responses, compared to their natural counterparts. For example, PEGylation―the covalent attachment of polyethylene glycol (PEG) chains to specific amino acid residues―has been employed to modify asparaginase, resulting in PEGylated asparaginase (pegaspargase; PEG-ASP), which demonstrates a significantly prolonged half-life and reduced immunogenicity in the treatment of acute lymphoblastic leukemia (ALL) [231,232]. Similar engineering strategies offer opportunities for enhancing the clinical performance of enzymes in anti-inflammatory therapeutics.

### 7.1. Directed Evolution of Tobacco Etch Virus (TEV) Protease

**Figure 8:**
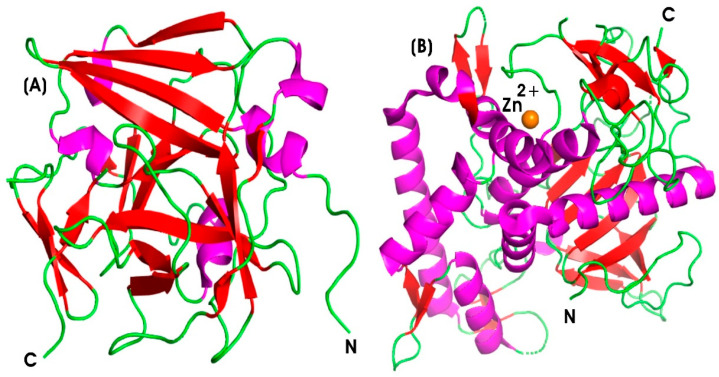
3D protein structures available in the protein data bank (PDB) for (**A**) native TEV protease (PDB ID: 1Q31) and (**B**) wild-type botulinum neurotoxin type A (BoNT/A) (PDB ID: 3NF3).

Tobacco Etch Virus (TEV) is a plant virus belonging to the Potyviridae family, primarily infecting tobacco and other plants and causing the tobacco etch disease. TEV protease, a highly specific cysteine protease with a molecular weight of 27 kDa, is encoded by the TEV genome. It plays a crucial role in the viral life cycle by canonically cleaving the viral polyprotein at a specific 6-amino acid sequence, “ENLYFQ”. Figure 8A illustrates the 3D protein structure of native TEV protease [233]. Due to its specificity and efficiency, TEV protease is extensively utilized in directed evolution to generate variants capable of cleaving specific peptides with enhanced catalytic activity towards novel substrates. 

Anti-inflammatory enzymes that degrade pro-inflammatory cytokines can mitigate the detrimental effects of chronic inflammation. In 2017, Liu and colleagues employed phage-assisted continuous evolution (PACE) to modify TEV protease to cleave a significantly different target sequence, HPLVGHM, found in the human pro-inflammatory cytokine IL-23. This engineering strategy represents a potential therapeutic approach for treating rheumatoid arthritis and psoriasis [234]. By altering the substrate specificity of TEV protease, the study provides insights into the development of enzyme-based anti-inflammatory therapeutics.

### 7.2. Protein Engineering of Botulinum Neurotoxin Type A (BoNT/A)

SNARE (soluble N-ethylmaleimide sensitive factor attachment protein receptor) proteins play a critical role in mediating the exocytotic release of inflammatory mediators, including vasoactive amines such as histamine, serotonin, and bradykinin [235]. Histamine contributes to increased vascular permeability and vasodilation, facilitating the migration of immune cells such as neutrophils and macrophages from the bloodstream into the inflamed tissues. Similarly, serotonin and bradykinin exacerbate inflammation by further increasing vasodilation and vascular permeability [236,237]. Mast cells utilize SNARE-dependent exocytosis to release histamine and other pro-inflammatory mediators from their granules, thereby amplifying the signs of inflammation, such as redness, swelling, and pain [238].

Botulinum neurotoxins (BoNTs), which are zinc-dependent metalloproteases and endopeptidases, are hydrolases produced by the bacterium Clostridium botulinum. They inhibit neurotransmission at the neuromuscular junction by cleaving SNARE proteins. Serotype A BoNT (BoNT/A), commonly known as Botox (~150 kDa), specifically cleaves the SNARE protein SNAP-25 (synaptosomal-associated protein of 25 kDa), thereby blocking synaptic vesicle fusion [239]. Figure 8B depicts the 3D structure of wild-type BoNT/A [240]. In inflammatory conditions, the secretion of vasoactive amines is predominantly mediated by the SNAP-25 isoform, SNAP-23. Despite the high sequence similarity (>75%) between SNAP-25 and SNAP-23, BoNT/A exhibits minimal activity towards SNAP-23 degradation.

In 2016, Binz and colleagues employed molecular dynamics simulations and mutational analyses to engineer the enzymatic domain of BoNT/A for improved SNAP-23 specificity. By substituting 10 residues of SNAP-23 with their SNAP-25 counterparts, they significantly improved its selectivity for SNAP-23 degradation with a 2-fold increase in catalytic efficiency, while concurrently decreasing its activity on SNAP-25 compared to the wild type. This novel-engineered BoNT/A variant can be a promising therapeutic agent for the treatment of diseases associated with SNAP-23-mediated hypersecretion of vasoactive amines and subsequent inflammatory conditions [241]. Further advancements in BoNT engineering have been achieved through phage-assisted evolution. In 2019, Blum and colleagues demonstrated the use of phage-assisted evolution to generate BoNT proteases with tailor-made specificities, enabling the rapid evolution of botulinum neurotoxin protease variants that selectively cleave novel targets, including proteins unrelated to their native substrates, while maintaining specificity and avoiding cleavage of non-target sequences [242]. These studies highlight the potential of BoNT engineering for developing next-generation protease-based therapeutics with precise anti-inflammatory applications while minimizing off-target effects. Overall, the directed evolution and engineering of proteolytic enzymes such as TEV protease and BoNT/A offer promising avenues for enzyme-based anti-inflammatory therapeutics. By enhancing substrate specificity and catalytic efficiency, these strategies pave the way for the targeted degradation of pro-inflammatory mediators, potentially leading to innovative treatments for chronic inflammatory diseases.

## 8. Conclusions and Future Perspectives

The development of enzymatic therapeutics has progressed alongside advancements in next-generation biomedicine. While the microbial production of anti-inflammatory enzymes has provided valuable therapeutic options, challenges such as immunogenicity have limited their widespread clinical use. As a result, human-derived enzymes have gained preference, particularly in enzyme replacement therapies, such as olipudase alfa for sphingomyelinase deficiency and avalglucosidase alfa for glycogen storage disorders. Recombinant enzymes produced via genetically modified organisms have also been explored; however, with the advent of biologics, including monoclonal antibodies and small-molecule drugs, enzyme-based therapeutics have seen a relative decline in clinical applications.

Recent advancements in anti-inflammatory enzyme production technologies, particularly using mammalian cell cultures such as Chinese hamster ovary (CHO) and human embryonic kidney (HEK) cells, offer promising avenues for generating human-like recombinant enzymes with enhanced stability, activity, and reduced immunogenicity. Combining enzyme engineering strategies with these production platforms can facilitate the development of highly efficient anti-inflammatory enzymes with optimized pharmacokinetic and pharmacodynamic properties. The combination of rational protein design, directed evolution, and post-translational modifications is crucial for overcoming challenges associated with enzymatic degradation, stability, and targeted therapeutic delivery. Moreover, innovative strategies for deimmunization and immunoevasion are required to enhance the therapeutic potential of non-human anti-inflammatory enzymes. For instance, epitope engineering has shown promise in reducing immune responses, as demonstrated by an engineered lysostaphin variant that elicited diminished antibody responses in human leukocyte antigen (HLA) transgenic mice [243]. However, further clinical evaluations are necessary to validate these findings. PEGylation remains a widely employed technique for reducing immunogenicity, extending half-life, and minimizing the rapid clearance of therapeutic enzymes. However, the emergence of anti-PEG antibodies poses a significant challenge, necessitating alternative approaches such as glycoengineering and site-specific conjugation strategies [239]. To address limitations related to immunogenicity, target specificity, stability, and therapeutic efficacy, the development of antibody-enzyme conjugates (AECs) represents a promising approach. These conjugates involve linking enzymes to antibody fragments (Fab), Fc regions, or full monoclonal antibodies (mAbs) to enhance targeted delivery and bioavailability. Notably, Palleon Pharmaceuticals has pioneered the development of E-602, a first-in-class engineered bi-sialidase-Fc fusion enzyme designed to degrade sialic acid-containing glycans on T-cell surfaces, thereby promoting immune activation. This innovative therapy is currently undergoing a phase II clinical trial for lupus nephritis [244].

In conclusion, overcoming key challenges such as immunogenicity, shelf-life stability, therapeutic efficiency, and cost-effective scalability through advanced protein engineering, rational design, and directed evolution strategies can unlock the vast therapeutic potential of both human and non-human anti-inflammatory enzymes. By addressing these critical aspects, enzymatic therapeutics for inflammatory diseases hold immense promise for revolutionizing modern biomedicine, offering safer and more effective treatment modalities for a wide range of inflammatory disorders.

## Figures and Tables

**Table 1:** List of therapeutic anti-inflammatory enzymes: names, brand names, manufacturer and mechanism of action.

S. No.	Enzyme	Brand Name	Manufacturer	Anti-Inflammatory Mechanisms	References
1	Catalase	1. Catalase-250, 2. CAT-200, 3. CATzyme	1. Creative Enzymes, Shirley, NY, USA, 2. Megazyme International Ireland Ltd., Wicklow, Ireland, 3. MP Biomedicals, LLC, Santa Ana, CA, USA	Converts ROS species (hydrogen peroxide) into water and oxygen, reduces oxidative stress in inflamed tissues, inhibits NF-κB activation, and decreases pro-inflammatory cytokines.	[64,65]
2	Superoxide dismutase	GliSODin, Orgotein	ISOCELL SA, Paris, France	Neutralizes ROS, reduces stress-induced inflammation, modules cytokine production, and inhibits NF-κB pathway.	[66,67]
3	Trypsin-Chymotrypsin	Enzomac, Combizyme,	Macleods Pharmaceuticals, Mumbai, India	Degrades pro-inflammatory mediators like fibrin at injury sites, promotes fibrinolysis, and aids in clearing necrotic tissues for faster healing.	[68,69,70,71]
4	Chymotrypsin	Orthomol Chondro	Macleods Pharmaceuticals, Mumbai, India	Works synergistically with trypsin to reduce tissue inflammation, clear inflammatory debris, and reduce edema and swelling.	[69,71]
5	Nattokinase	NSK-SD, Doctor’s Best Natto-Serra	Japan Bio Science Laboratory, Tokyo, Japan (NSK-SD) & Doctor’s Best, San Clemente, CA, USA (Natto-Serra)	Degrades fibrin clots, improves circulation, reduces inflammation, suppresses pro-inflammatory cytokines (IL-6 and TNF-α), and reduces oxidative stress and tissue damage.	[72,73,74,75,76]
6	Bromelain	Bromelain, Ananas	Nature’s’ way, Green Bay, WI, USA (Bromelain), NOW Foods, Bloomingdale, IL, USA (Ananas)	Inhibits COX-2 and reduces prostaglandin production, modulates cytokine levels (TNF-α, IL-1β), suppresses inflammation, reduces swelling and edema, and promotes fibrinolysis.	[16,77,78,79,80,81]
7	Papain	Accuzyme	Smith & Nephew, Inc., Andover, MA, USA	Facilitates wound debridement, treats chronic ulcers and burns, and reduces inflammation by breaking down necrotic tissues.	[82,83,84]
8	Serratiopeptidase	1. Serracore-NK, 2. Danzen, 3. Serra	1. Arthur Andrew Medical, Tucson, AZ, USA, 2. Takeda Pharmaceuticals, Tokyo, Japan, 3. Vitalzym, World Nutrition, Inc., Mesa, AZ, USA	Reduces inflammation and swelling, enhances tissue repair, reduces scarring, improves cardiovascular health, and inhibits bradykinin, which promotes inflammation.	[85,86,87,88]
9	Collagenase	Santyl (ointment)	Smith & Nephew, Andover, MA, USA & Worthington Biochemical Corporation, Lakewood, NJ, USA	Breaks down collagen in damaged tissue, reducing fibrosis and promoting tissue remodeling, clears necrotic tissues, aids in inflammation resolution, and facilitates the reduction of pro-inflammatory cytokines.	[89,90,91,92,93]
10	Hyaluronidase	1. Hylase 2. Hyalumax 3. Wydase	1. Riemser Pharma GmbH, Greifswald, Germany 2. Wockhardt Ltd., Mumbai, India 3. Wyeth Pharmaceuticals, New York, NY, USA	Breaks down hyaluronic acid in the ECM matrix, reduces tissue swelling, and enhances fluid drainage from inflamed areas.	[94,95,96,97,98]
11	Lysozyme	1. Lyso-6 2. Enzylex 3. Neuzym	1. Kora Biomedicine, Gyeonggi-do, Republic of Korea, 2. Omnix International, Dubai, UAE, 3. Nippon Zoki Pharmaceutical Co., Ltd., Osaka, Japan	Breaks down bacterial cell walls, reduces infection-driven inflammation, enhances phagocytic activity of immune cells, and modulates immune responses to decrease chronic inflammation markers.	[99]
